# A Software Framework for Remote Patient Monitoring by Using Multi-Agent Systems Support

**DOI:** 10.2196/medinform.6693

**Published:** 2017-03-27

**Authors:** Chrystinne Oliveira Fernandes, Carlos José Pereira De Lucena

**Affiliations:** ^1^ Department of Informatics Pontifical Catholic University of Rio de Janeiro (PUC-Rio) Rio de Janeiro Brazil

**Keywords:** eHealth systems, remote patient monitoring, biometric sensors

## Abstract

**Background:**

Although there have been significant advances in network, hardware, and software technologies, the health care environment has not taken advantage of these developments to solve many of its inherent problems. Research activities in these 3 areas make it possible to apply advanced technologies to address many of these issues such as real-time monitoring of a large number of patients, particularly where a timely response is critical.

**Objective:**

The objective of this research was to design and develop innovative technological solutions to offer a more proactive and reliable medical care environment. The short-term and primary goal was to construct IoT4Health, a flexible software framework to generate a range of Internet of things (IoT) applications, containing components such as multi-agent systems that are designed to perform Remote Patient Monitoring (RPM) activities autonomously. An investigation into its full potential to conduct such patient monitoring activities in a more proactive way is an expected future step.

**Methods:**

A framework methodology was selected to evaluate whether the RPM domain had the potential to generate customized applications that could achieve the stated goal of being responsive and flexible within the RPM domain. As a proof of concept of the software framework’s flexibility, 3 applications were developed with different implementations for each framework hot spot to demonstrate potential. Agents4Health was selected to illustrate the instantiation process and IoT4Health’s operation. To develop more concrete indicators of the responsiveness of the simulated care environment, an experiment was conducted while Agents4Health was operating, to measure the number of delays incurred in monitoring the tasks performed by agents.

**Results:**

IoT4Health’s construction can be highlighted as our contribution to the development of eHealth solutions. As a software framework, IoT4Health offers extensibility points for the generation of applications. Applications can extend the framework in the following ways: identification, collection, storage, recovery, visualization, monitoring, anomalies detection, resource notification, and dynamic reconfiguration. Based on other outcomes involving observation of the resulting applications, it was noted that its design contributed toward more proactive patient monitoring. Through these experimental systems, anomalies were detected in real time, with agents sending notifications instantly to the health providers.

**Conclusions:**

We conclude that the cost-benefit of the construction of a more generic and complex system instead of a custom-made software system demonstrated the worth of the approach, making it possible to generate applications in this domain in a more timely fashion.

## Introduction

### Innovative Technological-Based Solutions

Technological solutions can be applied to deal better with current operational problems involving the delivery of health care. The application of computational tools to hospital activities has the capacity to transform the present operational environment through activities such as improvement of work processes. Examples of improvements brought by the use of innovative technological solutions are as follows:

1. Change in the way the physician-patient–relationship occurs, because of Remote Patient Monitoring (RPM) possibilities [1]

2. Ease of information access and sharing among the medical team and the patients’ relatives [2]

3. More mobility for patients, whose health status can be monitored from home or work, without being restricted to hospital facilities

4. Possibility of collaborative work between the local team and external professionals; it allows a second opinion about patients’ diagnoses and treatments, as patient information is already in a distributed database

5. Possibility of automatic processes such as vital patient data collection by using sensors

6. Remote and real-time monitoring of patient health conditions

7. Alerts to health care professionals in emergency situations

8. Decrease in elapsed time for detection of anomalies in the vital signs of monitored patients, by using software agents; in this context, software agents consist of computational entities that perform activities in response to emergency situations

Investments in RPM technology can provide better support for patients from their health care team and perhaps make resources available for other health-related activities.

### Theoretical Background

#### Internet of Things (IoT)

IoT is a field within Computer Science that has grown quickly in recent years. Kevin Ashton introduced the term “Internet of Things” in 1999 [3]. One can define IoT as a global network of smart devices that can sense and interact with their environment for communication with users and other things (smart devices) and systems. In this context, things could be identified solely by using radio-frequency identification (RFID) [4] tags in order to be connected to the Internet and publish their information. Things are physical objects such as refrigerators, cars, walking sticks, dog collars, and whatever object comes to mind.

Thus, using sensors, actuators, and RFID-like technology, objects in the environment could be viewed, identified, and controlled more autonomously. In this case, things themselves could specify when they needed to be replaced, fixed, or report if they could provide data [3].

#### IoT Technologies: RFID, Microcontrollers, and Sensors

To develop the IoT patient-monitoring application described in this paper, 3 main IoT technologies have been used: RFID, micro-controllers, and sensors.

RFID is an automatic identification method that utilizes radio signals, recovering and storing data remotely through devices called RFID tags. These devices are used for identification, sensing, and communication [5].

**Figure 1:**
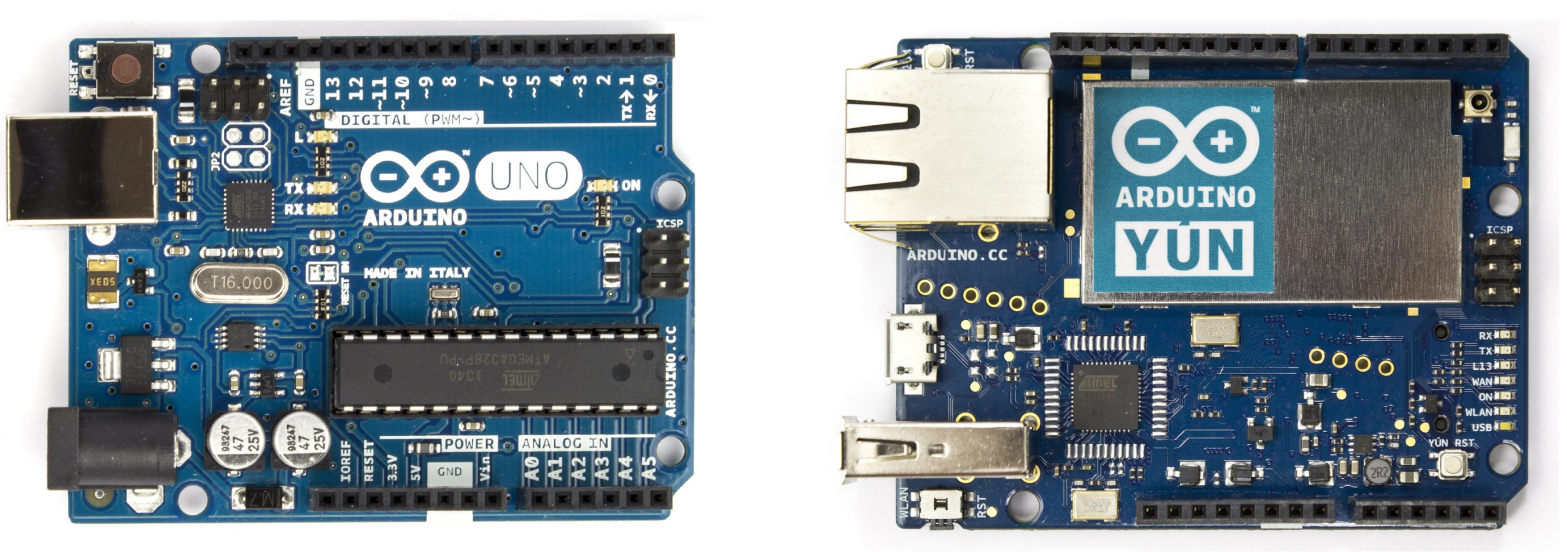
Arduino micro-controllers, Uno R3 (on the left) and Yún (on the right) models.

Arduino [6] microcontrollers, which are open source platforms for electronic prototyping, are also used: Uno R3 [7] and Yún [8] models (Figure 1). Microcontrollers can be programmed to process inputs and outputs of connected external components (Figure 1). One can use embedded computing to allow the construction of systems that interact with the environment using hardware and software [9].

A variety of sensors can be used to collect data for IoT applications such as temperature, humidity, light level, oxygen level, and sensor presence, among others.

**Figure 2:**
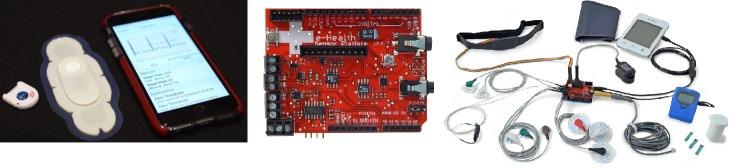
HealthPatch MD (on the left), e-Health Sensors Shield and e-Health Sensor Platform Complete Kit (on the right).

In eHealth, it is common for some devices to contain a number of sensors linked together, such as in the HealthPatch MD [10] Vital Connect health-monitoring sensor (Figure 2). The sensor is a small adhesive patch with a module that measures heart rate, breathing frequency, body temperature, posture, detection of falls, and also has Internet connectivity. Another example is the eHealth Sensor Platform Complete Kit [11]. It contains an eHealth Sensor Shield compatible with Arduino and Raspberry Pi [12] microcontrollers (Figure 2), plus 10 sensors to collect biometric data (Figure 2): pulse, oxygen levels in blood, airflow (breathing), body temperature, electrocardiogram (ECG), glucometer, galvanic skin response, blood pressure, patient position (accelerometer), and muscle or electromyography sensor (EMG).

#### Software Agents

A software agent [13] is an element of a computational system that is situated in an environment where it can perform autonomous actions in order to reach its assigned goals. An agent is both autonomous and capable of learning from its experience. Autonomy has been acknowledged as a key characteristic of an agent in satisfying its goals [14]. In this context, autonomy means operating without the intervention of humans or other systems, although the set of possible actions should be previously defined.

Although agents control their behavior and internal states, they do not have full control of the environment in which they operate. Agents contain a set of actions that can carry out tasks, the execution of which can result in changes in the environments. For this reason, one can consider that an agent can have partial control and influence over its environment depending on the action performed [15].

In general, the use of software agents is justified by the fact that asynchronous software systems require autonomous operation, a general argument that can be applied to our solution.

#### General Concepts About Software Frameworks

Frameworks are tools used to generate applications related to a specific domain; that is, to cope with a family of related problems [16]. The choice of using existing frameworks or developing new application generators is based on whether the framework can offer design and code reuse. Thus, frameworks can usually increase software development productivity and shorter time-to-market, compared with traditional approaches.

Frameworks contain fixed and flexible points known as frozen spots and hot spots, respectively. Hot spots are extension points that allow developers to create a new application from the framework instantiation process. In this case, developers should create specific application code for each hot spot, through the implementation of abstract classes and methods defined in the framework. Frozen spots consist of the framework’s kernel, corresponding to its fixed parts, previously implemented and hard to change. A frozen spot calls one or more of the application’s hot spots and is present in each framework’s instance [16].

Creating a new instance of a framework consists of 3 main steps: (1) Domain analysis, (2) Design, and (3) Instantiation. The domain analysis step includes requirements elicitation including definitions of hot and frozen spots. The design step is responsible for specifying the hot and frozen spots through a modeling language such as UML [17] diagrams. Design patterns [18] are also used in this phase. The instantiation phase corresponds to the application generation phase through hot spot implementation [16].

#### Related Work

Our proposal takes a similar approach to that in [19]. This paper shows the implementation of a distributed information infrastructure that uses the intelligent agent paradigm for: (1) automatically notifying the patient’s medical team regarding the abnormalities in his or her health status; (2) offering medical advice from a distance; and (3) enabling continuous monitoring of a patient’s health status. In addition, the authors have promoted the adoption of ubiquitous computing systems [20] and apps that allow immediate analysis of a patient’s physiological data such as a personalized feedback of their condition in real time, by using an alarm-and-remember mechanism. In this solution, patients can be evaluated, diagnosed, and cared for through a mode that is both remote and ubiquitous. In the case of rapid deterioration of a patient’s condition, the system automatically notifies the medical team through voice calls or SMS messages, providing a first-level medical response. This proposal differs from ours, in that the resulting application is closed, as opposed to our broader eHealth application generator.

The approach in [21] focuses on design and development of a distributed information system based on mobile agents to allow automatic and real-time fetal monitoring. Devices such as a PDA, mobile phone, laptop, and personal computer are used to capture and display the monitored data.

In [22], mobile health apps are proposed as solutions for (1) overcoming personalized health service barriers; (2) providing opportune access to critical information on a patient’s health status; (3) avoiding duplication of exams, delays and errors in patient treatment.

## Methods

### Main Research Goals

Our main research goal is to demonstrate that the formulation of a software framework to generate IoT applications in the eHealth domain does effectively support RPM. The aim is to analyze the tradeoffs involved in the challenge of building a flexible and powerful tool to help deal with the constraints found in a medical care environment. This initial version is totally experimental; it has not been tested in real medical care environments. Regarding the long-term goals of the research, the aim is to apply this software framework in a real medical care environment to assess its effective use as well as adequacy in terms of regulatory approval.

### Methodology

We decided to build an IoT framework to allow the characterization of the RPM domain by using framework design techniques that encompass software agents. Framework methodology was chosen to assess its suitability for the RPM domain and its potential to generate customized applications that achieve the stated goals of more closely connecting patients to their health care team. As a proof of the concept, 3 applications were developed with different implementations for each hot spot of the framework.

An application named, Agents4Health was selected to illustrate the instantiation process and the IoT4Health framework operation. Furthermore, IoT devices were built from scratch to collect patient data for the Agents4Health application by using hardware prototypes comprised of biometric sensors and Internet-enabled microcontrollers to send the sensed data to the cloud automatically.

To measure the ability of the tool to respond proactively to adverse conditions such as anomalies in patients’ vital signs, and its capacity to notify health providers in real time, the following step-by-step experiment was conducted:

1. Five measurement points were identified in the Agents4Health’s workflow related to the tasks performed by agents and were labeled as Timestamps (T1 through T5) as follows:

T1. The Agents4Health application retrieves the patient data from the cloud and the monitoring agent analyses them, searching for anomalies. If no anomaly is detected, the system remains in a loop collecting more data until an anomaly is found. Once an anomaly is detected the application continues to T2

T2. This second step is reached when the monitoring agent detects an anomaly and then calls the notification agent.

T3. The notification agent initiates the routine to notify the health care providers;

T4. The notification agent sends information about the detected anomaly to the patient’s health care providers;

T5. The health care providers receive the notification message on their mobile phones.

2. Agents4Health is executed and the timestamps are measured and registered.

3. Four delays defined as follows are captured for the different agent’s execution tasks:

Detection anomaly interval (DAI)=T2−T1. The anomaly's detection delay in the monitoring routine.

Notification start interval (NSI)=T3−T2. The delay between the anomaly detection and the initiation of the notification routine.

Notification period (NP)=T4−T3. Duration of the notification routine by agents.

Notification routine interval (NRI)=T5−T4. Time elapsed between the sending of the notification and its receipt by the health provider.

These delays were calculated to serve as a concrete measure of how quickly and proactively the solution can respond to the environment, as well as to support the assertion that this system performs anomaly detection in real time.

To confirm the fulfillment of the main research goal, the experiment described above was conducted and the relevant results have been tabulated in the Discussion section.

## Results

### IoT4Health Framework

#### Domain Analysis

In this step, problems that health professionals currently deal with in their patient monitoring routines are considered. As mentioned earlier in this paper, the decision was made to build a software framework instead of one or more apps. The choice to use framework design techniques was motivated by the fact that the construction of a more generic and complex system would provide a cost-benefit, in that frameworks can usually increase software development productivity and shorter time-to-market.

### IoT4Health Design

Regarding the IoT4Health’s design, besides the hot and frozen spots that were modeled as UML diagrams, the following architecture was defined.

### IoT4Health Architecture

**Figure 3:**
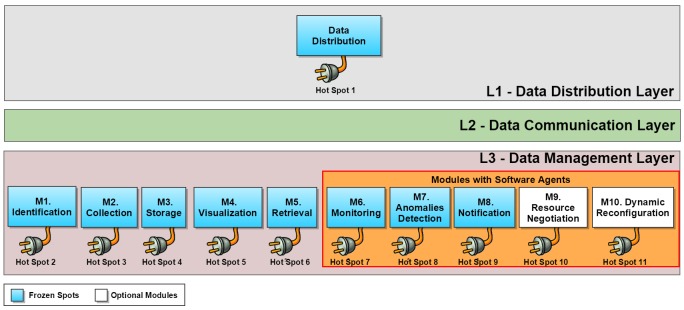
The IoT4Health’s architecture with its three layers (L1-L3). Data Management Layer (L3) and Distribution Data Layer (L1) interact through the Data Communication Layer (L2). The IoT4Health’s frozen spots can be extended by hot spots.

The IoT4Health’s architecture is structured in 3 layers, each with well-defined functionality (Figure 3).

**Figure 4:**
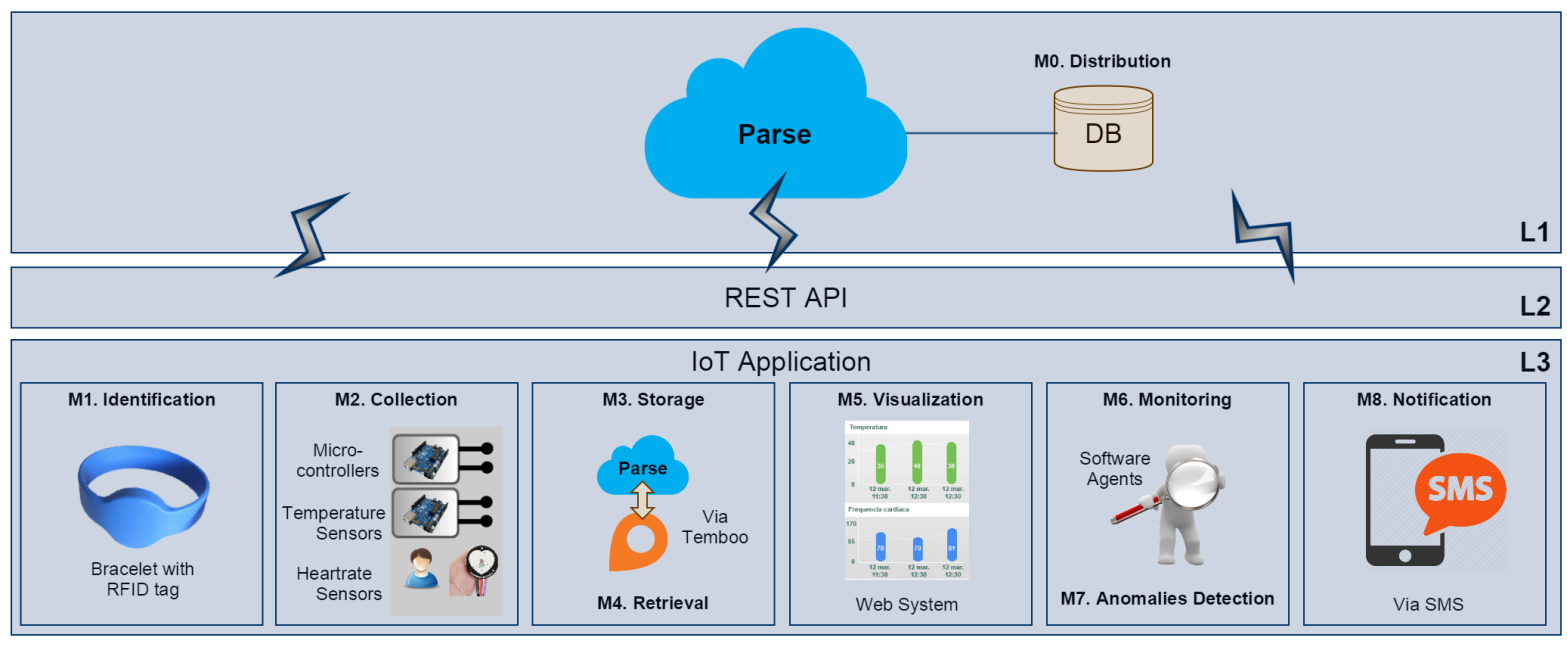
The Agents4Health’s architecture with its three layers (L1-L3). The Agents4Health application (L3) interacts with Parse (L1) through REST API (L2).

#### L1: Data Distribution Layer

This layer works as a remote database of a patient’s vital signs.

#### L2: Data Communication Layer

Through this layer, the L3 can communicate with the L1 layer’s remote database.

#### L3: Data Management Layer

The L3 Layer is responsible for the entire information management of the instantiated applications. It is composed of 10 modules with well-defined responsibilities. These modules are shown next, along with a description of their purpose and examples of how our framework can be extended by specific application code implemented in IoT4Health’s instances.

##### Identification Module (M1)

It should be implemented by IoT4Health’s instances to support the patient identification process. The IoT4Health’s architecture offers the possibility of customizing this process, allowing developers to use different strategies, including: (1) The use of a unique identification code, such as the patient’s ID Card; (2) utilization of RFID tags that can be inserted into objects like bracelets, cards, or other elements with radio-frequency identification capability; and (3) Biometry.

##### Collection Module (M2)

It provides the collection of both patient and his or her environment data. The collection process can be realized manually or automatically.

##### Storage Module (M3)

Its implementation lets the application store the vital collected data. Examples of storage strategies that can be developed as an extension of IoT4Health’s architecture are (1) Local storage, (2) Cached storage, and (3) Remote storage.

##### Visualization Module (M4)

It was designed to provide users with ways to visualize storage data. Developers can implement some visualization strategies, utilizing the Web or a mobile application.

##### Recovery Module (M5)

It is responsible for recovering patient data stored on the cloud-based platform.

##### Monitoring Module (M6)

It was designed to continuously monitor the sensed data through software agents. Agents evaluate if the sensed data are within normal ranges, thus monitoring them to find anomalous values (AV). These normal ranges are defined for each patient, accounting for age, gender, other individual patient conditions, and each sensor in use. The system has a mandatory configuration step for each patient that can easily be completed by an administrator filling out a form through a system interface. In this step, the following parameters are defined:

1. Desired value range (DVR): They are the normal values collected from sensors; that is, values within an acceptable limit. They can correspond to an interval such as 36.0-36.6 for body temperature, for example. They should be defined for each sensor in use.

2. AVs: They are values outside the DVR, which are associated with anomalies. Regarding the DVRs from the previous example, one could have 37.8 as an example for a temperature AV.

3. Label of Anomaly: They are associated with the AVs and must also be defined for each sensor. Regarding data like temperature, it can be associated with the following anomalies: (1) Hyperthermia, for example, can be the label specified for anomalies associated with AVs higher than 36.6; (2) Hypothermia, to AVs lower than 36.0.

As one can observe, each such anomaly will receive a meaningful label regarding the health care context, so that it makes sense to a domain specialist. The goal is to enable a health care provider to identify quickly what problem is occurring when the system has detected an anomaly.

##### Anomalies Detection Module (M7)

It is supported by the use of reactive agents. This entity triggers alerts to health providers when case anomalies are detected.

##### Notification Module (M8)

It offers the possibility of using different strategies to send alerts to the medical team, such as by short message service (SMS), email message, voice call, or by Bluetooth. This module also requires the configuration of some parameters, as follows:

1. Health provider responsible for an anomaly: a health professional should be selected to deal with each anomaly described.

2. Notification details: The type of message for each health care provider indicated previously should be specified (ie, SMS, email, voice call, or Bluetooth), along with the details such as email address or phone number.

This module’s result is the communication process between agents. Agents that monitor patient data send a message to agents that send notifications when they detect an abnormality in the patient’s condition, based on the predefined anomaly settings already mentioned.

##### Resource Negotiation Module (M9)

It utilizes the concept of cognitive agents that, in this context, would be responsible for the use of argumentation techniques [23] to achieve resource sharing in a collaborative way, by making its management more effective. An application could implement cognitive agents, responsible for adopting negotiation strategies, to obtain hospital resources for a particular patient.

##### Dynamic Reconfiguration Module (M10)

Its goal is to provide applications with context-sensitive capability so that these systems could be capable of responding to changes in the environment. A change of a patient’s room could affect the defined parameters for monitoring, anomaly detection, and notification modules, becoming inappropriate in the new context. In this case, the applications’ values must be reconfigured. This reconfiguration can be carried out manually by an administrator user or autonomously by cognitive agents.

### Frozen Spots and Hot Spots

The IoT4Health contains 11 hot spots, offering developers the opportunity to create customized applications. Each one of these modules has extension points that broaden our framework’s architecture, as shown above in Figure 3.

## The Application Agents4Health as an Illustrative Instantiation of IoT4Health

The Agents4Health application [1] is an example of the IoT4Health’s instantiation process, which was developed to illustrate the generative power of our framework Figure 4. It consists of a multi-agent system that autonomously conducts monitoring and notifying tasks. To access the patient data sensed by real biometric sensors and remotely stored through Arduino, the Agents4Health communicates with the cloud via REST application programming interface (API).

The Arduino integrated development environment (IDE) was used to implement the M2 and M3 modules of the Agents4Health in the C++ language. The other modules were created with the Java language. The software agents were programmed with the version 4.3.0 of the JADE tool [24]. JADE is a free software distributed by Telecom Italia (the copyright holder), in open source under the terms and conditions of the second version of the Lesser General Public License (LGPL) license. It is a framework to develop agent systems in Java. It simplifies multi-agent systems’ implementation through Foundation for Intelligent, Physical Agents (FIPA)-compliant middleware [25]. The JADE API offers 2 types of behavior classes that can be extended by agents: Primitive and Composite. The Agents4Health agents’ behavior was implemented using the Primitive behavior class. Each application agent is an extension of the *Agent* Class and has a corresponding behavior to *Behavior’s* extension class. The behavior of each of the system’s agents is defined by its setup method, where behavior was configured through the *addBehavior* method.

The Agents4Health application’s reactive agents present 2 types of behavior: *TickerBehavior* and *OneShotBehavior*. *TickerBehavior* type behavior is executed cyclically. That is, agents in our scenario that must carry out continuous monitoring activities implement this behavior, as is the case of *MonitoringSensorTemperatureDataAgent* class. Other agents are responsible for executing tasks that are not realized in a predefined interval of time, occurring only on demand in response to a specific event. This is the case of *NotificationBySMSAgent*, which sends messages to the medical team.

### The Agents4Health Instance

#### L1: Data Distribution Layer

To provide the data distribution service to the application, a remote data storage service called Parse [26] was utilized. However, because the Parse hosted service will be retired in early 2017, we are moving our database to another platform called MongoDB [27].

#### L2: Data Communication Layer

The Agents4Health communicates with Parse (L1) through the REST API [28]. The application sends and retrieves data to and from the cloud through HTTP requests.

#### L3: Data Management Layer

The data management layer comprises the IoT application, with its 8 modules (M1-M8) as follows:

##### M1

To identify a patient in the application, an RFID strategy has been chosen. We have used an RFID system that includes a tag and a reader. This process is performed through an RFID interface, where each patient receives a bracelet containing an RFID tag that will be used as a unique ID code in the system.

##### M2

**Figure 5:**
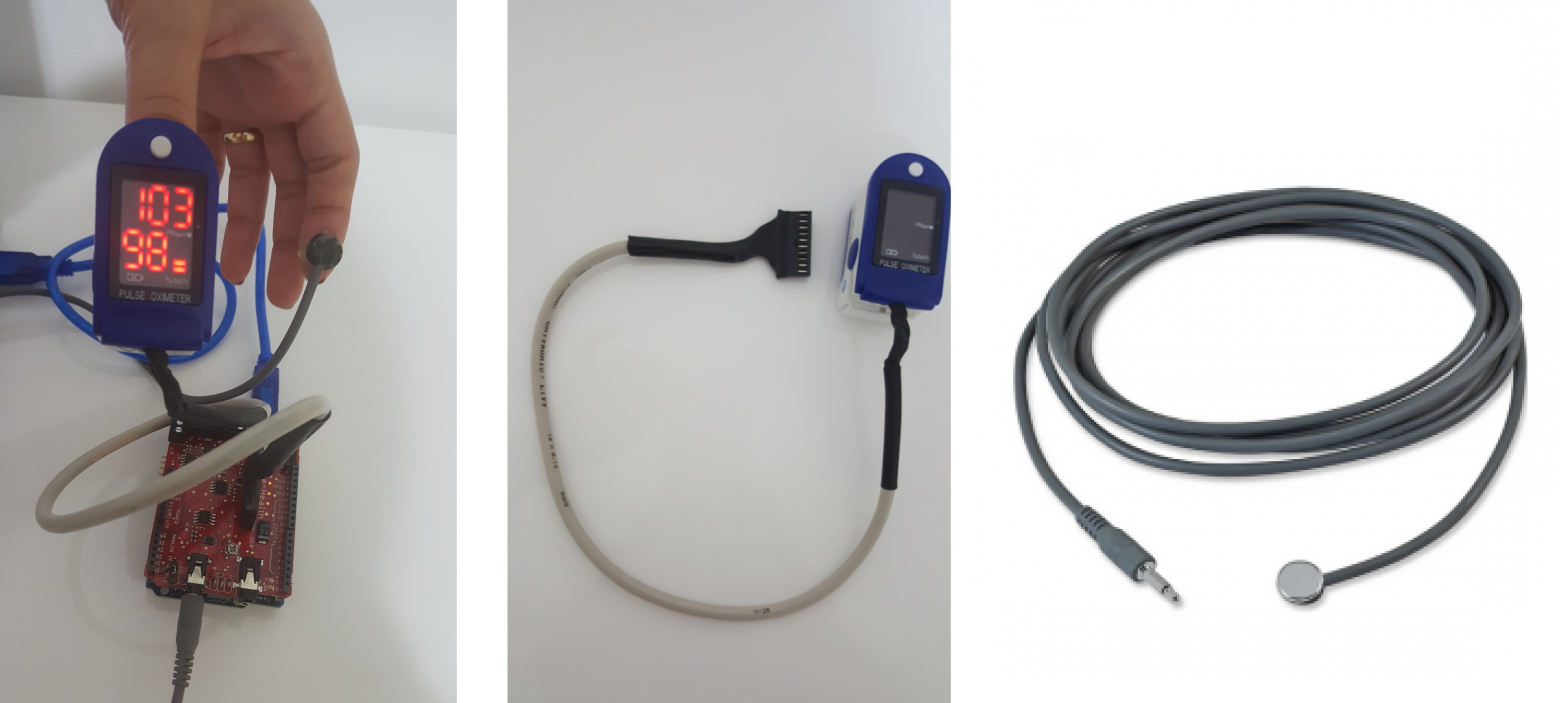
Our IoT device for patient monitoring that contains an Arduino microcontroller and biometric sensors (on the left), Pulse and Oxygen in Blood Sensor (SPO2) sensors and Body Temperature sensor (on the right).

In the Agents4Health application, both pulse and body temperatures are collected. This module may be extended to collect other patient data such as electroencephalography (EEG), EMG, as well as environment data such as light, noise levels, and data about the device such as battery status. This process, which is also called sensing, is performed automatically in Agents4Health. Arduino is used, together with sensors for heartbeat and temperature that form an IoT device capable of collecting patient data without human intervention (Figure 5).

##### M3

Once collected, the application transfers the patient’s data over the Internet to the Parse.

##### M4

The remote storage allows any authorized user to access the data by means of a user-friendly interface [1], through any device (computer, mobile phone, or tablet).

##### M5

**Figure 6:**
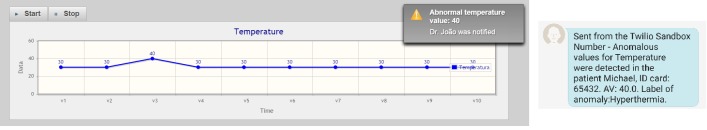
Example of the visualization module (on the left). Example of an action taken by the NotificationBySMSAgent agent (on the right).

A Web application is provided to support the visualization of the patient data. In the current implementation, there is a line chart for each one of the sensors used and they are updated in real time (Figure 6).

##### M6

Following the IoT4Health’s protocol, in this step, an administrative user defines the DVR and the AV for each sensor.

##### M7

During this phase, a specific label for each anomaly is defined for each of the sensors: heartrate sensor (Table 1) and temperature sensor (Table 2).

**Table 1:** Configuring an example for the anomaly detection module, considering cardiac heartbeat.

AV^a^ for cardiac heartbeat	Kinds of associated types of anomalies
Heartbeat values <60	Bradyarrhythmias such as sinus bradycardia or atrioventricular block
Heartbeat values >110	Tachyarrhythmias such as atrial fibrillation, supraventricular tachycardia, and ventricular tachycardia

^a^AV: anomalous value.

**Table 2:** Configuring an example for the anomaly detection module considering temperature.

AV^a^ for temperature	Kinds of associated types of anomalies
Temperature values <36	Hypothermia
Temperature values >36.6	Hyperthermia

^a^AV: anomalous value.

**Figure 7:**
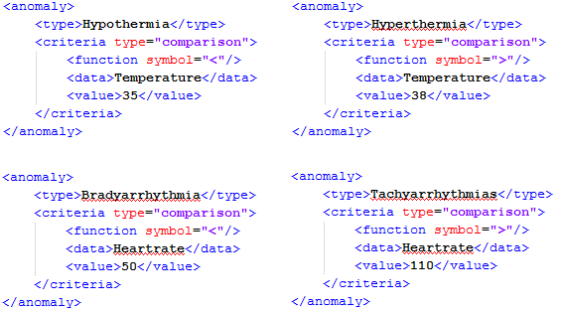
XML document that constitutes the agents’ knowledge base.

In Agents4Health, the criteria used by the reactive agents to detect anomalies are defined by the domain specialists and coded in the XML language. They will form the agents’ knowledge bases (Figure 7).

To prevent the system from detecting false abnormalities and triggering false alarms caused by simple patient movements or exercising, 2 strategies are being developed: (1) filtering the sensed data by using information provided by its own sensors related to the signal quality; (2) adding the environment’s sensors to collect information about the context of the measurement. The former is performed when the sensors in use provide information about signal quality. Sensors such as the Mindwave Mobile Headset (NeuroSky) [29] are used to collect EEG data to provide this type of information. In this particular case, if the sensor is not in contact with the skin or if there is some interference such as a strand of hair between the sensor and the skin, the signal quality will indicate this situation. In that case, the application can be configured to ignore the sensed data until the signal quality provides a reliable value. The latter is useful to make the AVs flexible, taking into consideration the context of the patient being monitored. To avoid mistakenly detecting a heartbeat anomaly, for example, when a patient is engaged in physical activity, we can use sensors such as an accelerometer to collect context information.

M8: For Agents4Health, the choice was to send SMSs as a notification strategy, using the Twilio [30] library. Twilio is a platform using API communication that offers Web-service APIs, allowing users to construct their own SMS communication applications.

## Discussion

### Conclusions and Future Work

The main objective of this paper was to report on the construction of the IoT4Health framework based on a requirements analysis of the RPM domain. This was accomplished by using framework design techniques and the introduction of software agents in the framework design to allow autonomic behavior [31]. To deal with the aforementioned goal, experiments for the IoT4Health framework were created and tried.

In a main application experiment, generating a complete instance of the framework validated IoT4Health. This experiment, called Agents4Health, made it possible to observe that its design contributed toward making the patient’s environment more proactive. Through this experimental system, it has also been possible to detect anomalies in real time and to send alerts instantly and autonomously to health providers. Thereby, professionals responsible for taking action in the case of abnormalities in a patient’s condition can immediately react to these events.

As mentioned in the Methods section, Table 3 offers the results of the experiment conducted to offer a more concrete way to indicate the performance of the simulated environment:

**Table 3:** Examples of timestamps for agents’ behavior and task delays.

Timestamp T1	Timestamp T2	Timestamp T3	Timestamp T4	Timestamp T5	DAI^a^ (s)	NSI^b^ (s)	NP^c^ (s)
2016-11-04-173424	2016-11-04-173427	2016-11-04-173427	2016-11-04-173428	2016-11-04-1734	3	0	1
2016-11-04-173430	2016-11-04-173434	2016-11-04-173434	2016-11-04-173436	2016-11-04-1735	4	0	2
2016-11-04-173442	2016-11-04-173445	2016-11-04-173445	2016-11-04-173448	2016-11-04-1735	3	0	3
2016-11-04-173450	2016-11-04-173454	2016-11-04-173454	2016-11-04-173455	2016-11-04-1735	4	0	1
2016-11-04-173458	2016-11-04-173501	2016-11-04-173501	2016-11-04-173502	2016-11-04-1735	3	0	1
2016-11-04-173504	2016-11-04-173507	2016-11-04-173507	2016-11-04-173509	2016-11-04-1735	3	0	2

^a^DAI: detection anomaly interval.

^b^NSI: notification start interval.

^c^NP: notification period.

On average, the DAI for the Agents4Health experiment results is 3.5 s. The NSI presented zero delays for all results in this experiment. The NP averaged 1.75 s. And, finally, as mobile phones do not provide the SMS reception time with millisecond precision, there is only an approximate measurement for NRI, which was less than 1 min on average.

We have been involved in a number of practical developments based on our framework. One consists of the use of Bluetooth for communication with the medical team in the absence of Internet access. The use of machine learning is also examined in the patient monitoring domain. This approach is performed by creating melanoma and mammography classifications as a black box accessible to the system agents. IoT4Health is also being used as the basis of a complex patient monitoring system under development in our laboratory [32], with our participation and which has been named portable care.

As future work, we are planning a rigorous formal characterization of the patient monitoring domain as well as the formal characterization of the family of applications reachable through the framework flexible points. The application of cognitive agents as elements of the software framework is also being considered.

## Abbreviations

API: Application Programming Interface
AV: anomalous values
DAI: detection anomaly interval
DVR: desired value range
ECG: electrocardiography
EEG: electroencephalography
EMG: electromyography
FIPA: Foundation for Intelligent Physical Agents
HTTP: Hypertext Transfer Protocol
IDE: integrated development environment
IoT: Internet of Things
JADE: Java Agent Development Framework
NP: notification period
NRI: notification routine interval
NSI: notification start interval
RFID: radio-frequency identification
SMS: short message service
UML: Unified Modeling Language
XML: eXtensible Markup Language
